# Structure analysis of group I plant nucleases

**DOI:** 10.1107/S0909049510030700

**Published:** 2010-11-05

**Authors:** Jan Dohnálek, Tomáš Koval’, Petra Lipovová, Tomáš Podzimek, Jaroslav Matoušek

**Affiliations:** a Institute of Macromolecular Chemistry AS CR, v.v.i., Heyrovského nám. 2, 162 06 Praha 6, Czech Republic; b Institute of Physics AS CR, v.v.i., Na Slovance 2, 182 21 Praha 8, Czech Republic; c Faculty of Mathematics and Physics, Charles University in Prague, Ke Karlovu 3, 121 16 Praha 2, Czech Republic; d Institute of Chemical Technology, Technická 5, 166 28 Praha 6, Czech Republic; e Institute of Plant Molecular Biology, Biology Centre, AS CR, v.v.i., Branišovská 31, 37005 České Budějovice, Czech Republic

**Keywords:** nuclease, protein structure, crystal structure, glycosylation, anticancer drugs

## Abstract

Anticancer drugs attacking nucleic acids of the target cells have so far been based on animal or fungal ribonucleases. Plant nucleases have been proved to exhibit decreased cytotoxic side effects. Tomato bifunctional nuclease 1 with activity against both single-stranded and double-stranded RNA and DNA was produced in tobacco leaves as recombinant protein. The enzyme crystallizes under several different crystallization conditions. The presence of Zn^2+^ ions was confirmed by X-ray fluorescence. First crystallographic data were obtained.

## Abstract 2

Structural properties of plant nuclease TBN1 are studied using synchrotron radiation to explain its specificity, role of glycosylation and to contribute to potential application in cancer treatment.

## 1. Nucleases as anticancer drugs

DNA and RNA degrading enzymes from mammals, animals and fungi have been intensively studied for their activity in degrading tumor cells. Since 1938, when bovine pancreatic ribonuclease was isolated for the first time by René Dubois, efforts have been made to utilize the cytotoxic activity of ribonucleases (RNases) in the fight against cancer (Matousek, 2001 ▶; Ledoux, 1955*a* ▶,*b* ▶; Deonarain & Epenetos, 1998 ▶). It is both their natural regulatory function in gene expression as well as high cytotoxicity which make them attractive targets for biochemical, structural and mutagenesis studies. A number of RNases have been successfully used to suppress and kill tumor cells including bovine pancreatic ribonuclease (RNase A), α-sarcin, bovine seminal ribonuclease, onconase (ONC) and human-eosinophil-derived neurotoxin (Ledoux, 1955*a* ▶,*b* ▶; Ledoux & Baltus, 1954 ▶; Alexsandrowicz, 1958 ▶; Better *et al.*, 1992 ▶; Wool, 1997 ▶; D’Alessio & Leone, 1963 ▶; Darzynkiewicz *et al.*, 1988 ▶).

The interaction of these enzymes with cells is mostly non-specific with respect to the cell type and in many cases their application causes strong side effects, such as changes in metabolism, immuno-reactions or interference with spermatogenesis (Matousek, 1973 ▶, 1975 ▶; Soucek *et al.*, 1996 ▶; Matousek *et al.*, 2003 ▶; Matousek & Matousek, 2010 ▶).

## 2. Potent plant nucleases

Recently, investigations began into the applicability of plant nucleases in interference with tumor or virally infected cells. Higher plants offer nucleases and ribonucleases that are as yet functionally and structurally not sufficiently characterized, some of which promise weaker side effects and efficient anti-tumor activity. The reasons for this could lie in their glycosylation patterns and relative genetic distance from the affected organism (Matousek & Matousek, 2010 ▶; Matousek *et al.*, 2010 ▶).

Bifunctional nuclease from *Solanum lycopersicum* [red tomato (TBN1, UniProt sequence accession No. AM238701)] belongs to the plant nuclease I family. Enzymes from this family share some common attributes: Zn^2+^, Mg^2+^ or Ca^2+^ dependence, ability to cleave RNA and denatured DNA much more efficiently than native DNA with 5′-mononucleotides as products, and their relation to fungal P1 and S1 nucleases. They are EDTA-sensitive, their pH optimum is in the range 5.0–6.5, and their molecular weight in the range 31–35 kDa. They also often play a significant role in many crucial parts of the plant life cycle including the development of tissues and organs, stress response and apoptosis. It has been shown that TBN1 plays a considerable role in specific apoptotic functions, tissue differentiation, vascular system development and response to viral pathogens (Matousek *et al.*, 2007 ▶).

Many nucleases from this family have similar structural features to animal ribonucleases (Chang & Gallie, 1997 ▶; Dangl *et al.*, 2000 ▶), *e.g.* a disulfide bridge playing an important stabilization role in BS-RNase (Wool, 1997 ▶). There is very little known about the mechanism of the activity of these very potent and promising endonucleases.

## 3. Towards the three-dimensional structure of TBN1

TBN1 was successfully produced in tobacco leaves by recombinant techniques. The purification procedure including precipitation and two chromatographic steps yields sufficient amounts of pure enzyme for structural studies (Matousek *et al.*, 2007 ▶; Koval *et al.*, 2009 ▶). The enzyme (molecular weight = 37 kDa) without the N-terminal signal sequence is N-glycosylated at three sites. For structural studies an initial crystallization screening was carried out (Index screen, Hampton Research, Aliso Viejo, CA, USA) using the hanging-drop vapour-diffusion method. The enzyme crystallized under several different crystallization conditions. Diffraction data of limited resolution and quality can be obtained.

**Figure 1:**
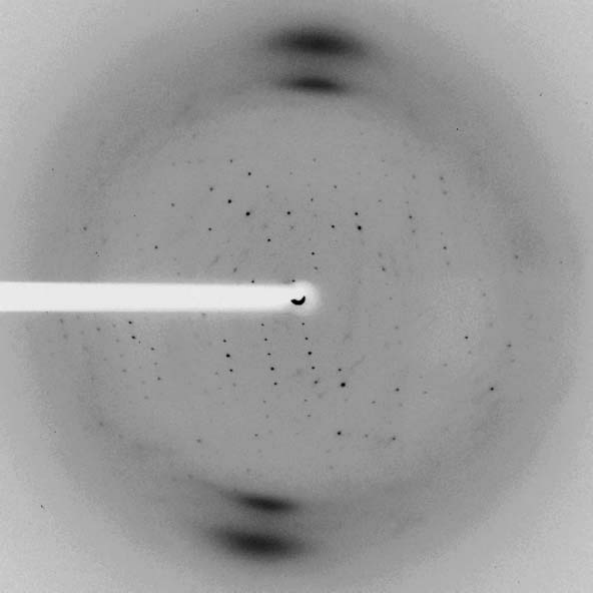
Anisotropic diffraction pattern collected from a crystal of tomato nuclease 1. The highest resolution reflections can be observed at about 3.6 Å.

Crystals from 0.1 *M* Bis-Tris pH 6.5, 10% *w*/*v* PEG 5000 MME and 4% *v*/*v* dimethyl sulfoxide, in space group *H*3, with unit-cell parameters *a* = *b* = 115.8 Å and *c* = 75.2 Å, were of variable diffraction quality. Testing of the crystals along with dataset collection was performed at beamline BL14.1 of the BESSY II synchrotron radiation source using a MARmosaic CCD 225 detector and a mini-kappa goniometer (Fig. 1 ▶). The presence of the natively bound Zn^2+^ ions was confirmed by an X-ray fluorescence spectrum measurement. To minimize the effects of fast radiation damage to these crystals the first data set to obtain sufficient anomalous signal was collected slightly above the experimentally determined Zn^2+^ absorption edge (λ = 1.2782 Å). One ω-scan dataset was collected (Δω = 1°, κ = 0, *t* = 10 s, 180 images) and processed using *HKL2000* (Otwinowski & Minor, 1997 ▶). The data, with diffraction limit 3.6 Å, *R*_merge_/*R*_merge-outershell_ = 0.10/0.54 and overall *I*/*σ*(*I*) = 18.3, were used for initial structure solution computations. Three zinc ions were localized using the *SHELX97* program package (Sheldrick, 2008 ▶), but the overall data quality, including anisotropy, disables structure solution. Approximately one-sixth of the molecular mass of the enzyme is formed by covalently attached oligosaccharides, which probably leads to uneasy crystallization and problematic diffraction data analysis.

TBN1 was proved to contain Zn^2+^ ions and its glycosylated form is crystallizable. The initial crystallographic analysis leads to clear identification of three zinc ions, which are expected in the enzyme active site, but does not provide phases sufficient for structure solution. The search for new crystal forms is in progress and variants of the enzyme and partially deglycosylated forms are being employed to improve crystallization properties.
